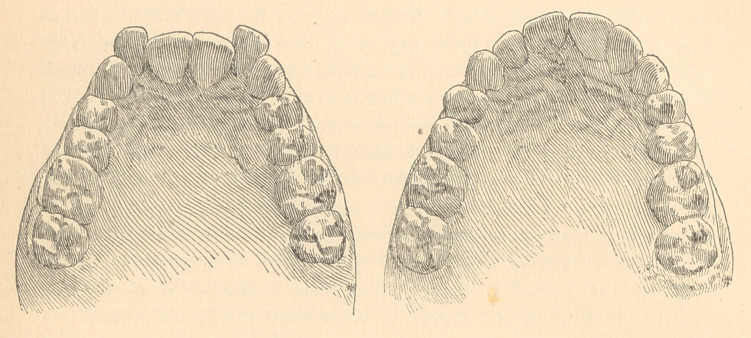# A Study in the Regulation of Teeth

**Published:** 1892-01

**Authors:** Rodrigues Ottolengui

**Affiliations:** New York


### A STUDY IN THE REGULATION OF TEETH.[1]

BY RODRIGUES OTTOLENGUI, M.D.S., NEW YORK.

Mr. President and Gentlemen,—Let us consider the position of a young graduate when the first case requiring regulation is presented to him. With no previous practical experience, can he rely upon the directions offered by text-books, and, by following them, feel assured of success? Unhesitatingly, I say, No! It

---

[1] Read before the Odontological Society of Pennsylvania, November 14, 1891.

is my object in this paper to show wherein the descriptions of cases in published reports are inadequate to guide the non-expert.

It has been the custom of writers first to consider etiology. This is science, and therefore admirable from that stand-point, but only in a few instances does it assist the practitioner who is about to undertake a case. For instance, if it can be determined that a given disfigurement is hereditary, it is at once indicated that the retaining-plate must be worn for a long time. But the teeth must be regulated first, before a retainer is even to be considered.

Next, we find the author giving a general description of methods, all of which is invaluable, but does not yet indicate the specific plan best adaptable to the case in hand. Lastly, we come to cases from practice. This is the most valuable knowledge offered; but even this is inadequate, for we find the author explaining how he accomplished results, without giving in detail the obstacles which occurred unexpectedly, and how they were overcome by special apparatus invented by the ingenuity made requisite by the necessities of the condition.

Notwithstanding the fact that we have already so many text-books, I believe that there is room for one more, which shall be written as I mean to write this paper,—that is to say, after one or two very brief chapters upon generalities, and a reference to other works for the science of the subject, to proceed by a continuous history of numerous cases from practice, well illustrated, giving failures as well as successes, and placing in juxtaposition the accounts of cases apparently similar, which nevertheless demanded totally different methods.

Without further preamble I will describe the one case which I have brought with me, and which I have denominated a study. Last October a young woman, aged eighteen, came to me with her teeth as shown in the models. Just here let me say that the best set of models procurable are not always sufficient in diagnostication. This case is one of those where the study of models alone will not lead to the correct solution of the problem. Yet this was the very error into which I fell. Let us, then, consider the models a moment, and I will explain the deductions upon which I at first began work. At a casual glance it appears that only four teeth are involved in the irregularity. The superior central incisors are tipped inward, while the laterals project forward. This forward projection of the laterals is seen to be more apparent than real. Were the centrals in normal position, it would simply be one of a common class of cases where the anterior tips of the laterals slightly overlap the centrals. The occlusion with the lower jaw being good, my first study of this case went no further than this; and I determined to begin by bringing the central incisors outward, after which the laterals could be slightly drawn back, and the case considered completed. To do this a very simple appliance was needed,—a vulcanite plate to cover the roof, held in place by clasps, and having a gold hook about opposite the sixth-year molar on each side. Such a plate in position, a rubber band could be caught over one hook, brought forward between the lateral and cuspid, then back between the lateral and central, forward again between the next central and the lateral, and backward around the other lateral, being finally caught over the other hook. The tension would tend to draw in the laterals and throw out the centrals by one action. I made this plate before again seeing my patient, but I never used it. Why? Because further study showed that more was required in this case than the movement of four teeth. A closer scrutiny of the face showed that an otherwise comely countenance was really spoiled by a retreating chin, which was apparently augmented by the protrusion of the upper lip. This made a total change of plan expedient. In the first instance the alteration was to have been accomplished by considerable forward movement of the central incisors, and only a slight retreating of the laterals. By this plan I would have made no change in the upper lip, but rather the contrary, since I should have sent outward two more teeth to support it. If I wished to lessen the protrusion of the upper lip, it became necessary that the greatest movement should be in the lateral incisors which should be moved backward, and the centrals brought forward only far enough to give them a more erect position. As to the retreating chin, that fact made an entirely new plan possible. It will be seen that in carrying the centrals forward, as first decided, there would then occur sufficient space for retreating the laterals. But if I were to undertake to bring the laterals backward first, there would be no room whatever for its accomplishment, all the teeth being present and closely contiguous. Space for this could be procured in either of two ways,—by extraction, a method reluctantly to be resolved upon, or by expansion of the arch. In this instance the occlusion would thus be destroyed, and sufficient expansion would leave the entire upper jaw, after regulation, biting outside of the lower, a sufficient cause, ordinarily, for not choosing it, submitting in preference to the loss of two teeth. With the disfigurement of a retreating chin, however, the case assumed a different aspect, for, after regulation of the superior jaw, the process known as jumping the bite would not only alter this defect in beauty but would restore a good occlusion. For these reasons the first method was abandoned, and widening of the arch was decided upon as a preliminary step.

Now, were I to follow the custom of authors, I could say here simply, a plate was made, supplied with a jack-screw, and the jaw was widened in so many days. The kind of plate and the jack-screw itself being well known, it might appear that I had said all that was necessary. I have seen various devices for widening an arch pictured and described, but I have never seen the difficulties of their application explained. Thus, when the young dentist confidently attempts to widen an arch with a jack-screw, he may be disgusted to find that he cannot do it. In my experience there have been very few cases where the jack-screw can be successfully used without special knowledge of how to overcome obstacles which will arise, or the ability to extricate one's self by ingenuity of invention. It may be advantageous, therefore, to say a few words about the jack-screw in connection with the plate.

Given a jaw not over fourteen years of age, in which the molars and bicuspids are well developed and of good size, standing in erect position so that their palatal sides are on parallel planes, the one side of the mouth with the other, and the widening of that jaw will be a pleasure. How often will such a condition be found in a mouth which is abnormal, since regulation is required? Any departure from these conditions adds to the difficulty of using the jack-screw and split-plate. The plate is made and allowed to cover the palatal surfaces of the posterior teeth for ever so slight a space, and what do we find? A plate which has a bevelled edge to it. As soon as the jack is tightened the tendency will be to throw the plate down towards the masticating surfaces of the teeth. The jaw cannot be widened with such a fixture. The remedy is to carefully trim away the plate till it meets the teeth, presenting a sharp edge. But suppose that after this is done it is still found that the plate is thrown down. The reason will be that the palatal surfaces of the teeth present slanted planes. One way to obviate the slipping is to make a new plate having spurs of gold to extend between the teeth so that they will catch under the knuckle.

I made what I deemed would be a suitable plate for this case. It was of black rubber, supplied with clasps, and well-fitted, the edges being sharp. When placed in the mouth and the screw tightened, it was very firm. The first result of using a jack-screw is always gratifying. The measurement with a compass will show considerable movement within one or two days. After that, progress is slower. This is because at first the teeth move in their sockets, as when separating for filling. After a week my patient complained very much of pain produced by the plate along the palatal wall. Examination showed that the gum tissue had been destroyed till the bone itself was in sight. This was deplorable, but how had it occurred? The patient being eighteen years old, the teeth were somewhat resistant, but the main cause was the fact that upon that side the teeth tipped slightly towards the palate, so that the tendency of the plate, under the pressure exerted by the screw, was to slide up against the gum, thus making pressure against soft tissue, which, of course, yielded and was lost. This plate, therefore, it was necessary for me to abandon. I made a new one similar in every respect to the other, but in addition supplied with a means of preventing a recurrence of the disturbance. Taking a bit of gold wire and burning the ends till they balled up, I fashioned it so that it rested across the sulci of the first and second bicuspids. Another bit of wire soldered to this, forming a T, passed down and entered the vulcanite. Each side of the plate being supplied with a T, the arms of which rested in the sulci of the bicuspids, it is plain that the plate could not be forced upward against the soft tissue. This proved a successful scheme, and the jaw was widened without further difficulty.

Before the expansion was completed, a second plate was requisite, because the first one became too much straightened under the action of the screw. This second plate was exactly like the last described, except that in addition there was a gold hook opposite the molar upon each side. As some space had been obtained by the widening which had been accomplished by the first plate, it was decided, when making the second fixture, to begin the retraction of the laterals. The plate in position, an elastic band was stretched from each hook over the lateral upon its own side.

At the end of the sixth week the jaw had become as wide as was deemed necessary. A comparison of the models will show that the asymmetry has been marvellously well corrected. It is also noteworthy that this patient is a gifted singer under the tutelage of one of the finest masters in the metropolis. Her lessons were necessarily interfered with during the regulating, which caused a deep growl of disapproval from the professor. After the regulation was completed this growl was changed into a song of praise, for the teacher asserts now that the added tongue-room has given the lady a richer tone, a better pitch, a more extended scale, and more control of her notes. Before passing from this I would say that the widening here gained in six weeks may seem inadequate to the time expended. It must be remembered that the patient was eighteen years of age. With children of twelve to fourteen I have seen a greater gain in two weeks, and in one instance but six days was required to widen a jaw the width of half a molar, more than which is rarely required. In that case the suture opened.

Returning to the case under consideration, we now come to the point where the plates for widening the arch were to be abandoned. The new fixture required was a simple plate to cover the roof of the mouth and fit accurately the palatal surfaces of the teeth. It was made stiff enough to resist the tendency of the jaw to resume its original form, and it was supplied with four hooks,—two opposite the molars as before, and two in the vicinity of the cuspids. A single rubber band was hooked to the posterior hooks and placed, as was intended in the first plate which I described and which was abandoned. To apply the band it was stretched across from hook to hook, and the plate placed in the mouth. Then a ligature was passed through one loop of the ligature, which was then brought forward till it rested over the four incisors. Next, the central part of the ligature was carried backward again and allowed to rest along the palatal surfaces of the centrals. The action would tend to force the centrals outward as well as bring in the laterals. But it was designed to reduce the laterals more, and therefore the other pair of hooks became necessary. A ligature was extended from each over the lateral on the opposite side of the mouth. With this the completion of the work was attained in two weeks more, making eight weeks in all the time spent upon the case up to this point.

The next step was to make a retainer, and the one made is seen upon one of the models. I have seen it stated by one author that a "cage" can be made of piano wire in fifteen to thirty minutes which will fit accurately. It is possible that we do not all mean the same thing when we say that a fixture fits. I wish to call attention to the "fit" of this appliance. The model shown was made from a plaster impression taken with the fixture in place. Before pouring, the gold appliance was placed in the impression, and you see the result. I have not attempted to separate it from the model, because the backings for the two centrals were made to extend under the gum, and therefore are tightly held in the plaster. Besides, were it removable from the model, there would occur an abrasion of the plaster teeth which would soon mar the appearance of fitting. It is noticeable that, while this cage is accurately adapted at certain points, there are one or two places where the band does not rest against the teeth; but this is only at points where absolute fit was not needed. A retainer of this kind is very cleanly, being removable, does not fill up the mouth, and can be made to hold teeth rigidly in almost any position, provided the teeth are long enough, or of such shape that it may be fashioned so that it will keep its place. I will explain how this is made, for there is nearly ten hours' work in the construction of such a piece, at least in my hands. First, to an accurate model made from a plaster impression half round clasp-gold wire is bent into clasps for the molars and for the second bicuspids. These are perfected by fitting about the natural teeth, and made to bind the teeth near the gum-line. Impressions are taken with them in place, and models poured with plaster and sand. These models will present the clasps in desired relation to the teeth, and they are united with solder. This process is repeated, going to the mouth and taking new impressions as often as may be requisite till the cage is completed. Ordinarily there will be no need for a backing to any teeth such as I have for the centrals, a simple bearing of the band being sufficient. But while a band will restrain teeth which have been retracted, as it does the laterals in this case, it will often fail to control teeth which have been sent outward. This is because the palatal surface is an inclined plane, and the action of the tooth, in its tendency to return to its original position, will simply throw down the fixture. To make the backing, make an accurate model and then carve away the gum, exposing the bulb at the neck of the tooth, carving it into shape. Make dies and swage the piece of pure gold plate. This will give an approximate fit, and the purity of the gold allows of its being readily burnished to the natural tooth, so that it is a perfect adaptation, extending below the gum and reaching a point of anchorage under the bulbous portion. The cage is then placed in the mouth binding the backing to place, and an impression and models made as before. Solder then unites the piece and stiffens the backing at the same time.

A fixture of this kind could only serve temporarily in this case, because it was planned to jump the bite. For this reason it would not have been made, except that the young lady wished to return to her home in Kansas for the Christmas holidays. She remained away for a month, and upon her return I made a plate with which to accomplish the jumping of the bite. This was a black rubber plate fitting accurately the back of the centrals, so that it should retain them, and supplied with an inclined plane, over which the lower teeth would be forced to reach. The laterals were controlled by a band around the labial surfaces. This biter was worn for four months, a longer term than is usually necessary. The habit being formed, a retainer was made which spanned the arch, two hooks passing outward over the posterior corners of the laterals, which were made to hold these teeth in their retracted positions. The final models show the result.

The points of special interest in this case are,—

*First.* It is one of a numerous class of irregularities, in which the best result is obtained after moving several teeth, notwithstanding the fact that the first study seems to show that but a few are involved in the irregularity.

*Second.* So simple a thing as the widening of the arch could only be attained by devising means of overcoming an unexpected difficulty in the abrasion of the soft tissue of the roof.

*Third.* So universal a retainer as the one shown could only serve a temporary purpose, because of the closeness of the occlusion.

*Fourth.* The unusual advisability of destroying a good occlusion and reconstructing it, because of the gain in facial symmetry and the advantages of voice-power obtained by slightly increased width of the arch. It is a question of some interest whether the improvement in singing was due to increase of tongue-room or to the improved curve of the arch.

*Fifth.* The length of time required to jump the bite, occasioned by the fact that either the age or the intense habit made the patient so reluctant that the inclined plane tended to throw the lower incisors outward rather than to alter the swing of the jaw. This spreading of the laterals made it necessary to abandon the inclined plane from time to time that the teeth might return to normal position.

*Sixth.* The beautiful result, which has repaid all the thought and work expended upon the case.

## Figures and Tables

**Figure f1:**